# The effect of COVID19 pandemic restrictions on an urban rodent population

**DOI:** 10.1038/s41598-021-92301-0

**Published:** 2021-06-21

**Authors:** Miguel A. Bedoya-Pérez, Michael P. Ward, Max Loomes, Iain S. McGregor, Mathew S. Crowther

**Affiliations:** 1 Brain and Mind Centre, The University of Sydney, Sydney, NSW 2050 Australia; 2 School of Psychology, The University of Sydney, Faculty of Science, Sydney, NSW 2006 Australia; 3 School of Life and Environmental Sciences, The University of Sydney, Faculty of Science, Sydney, NSW 2006 Australia; 4 Sydney School of Veterinary Science, The University of Sydney, Faculty of Science, Sydney, NSW 2570 Australia; 5 Lambert Initiative for Cannabinoid Therapeutics, The University of Sydney, Sydney, NSW 2050 Australia

**Keywords:** Urban ecology, Population dynamics, Invasive species

## Abstract

Shortly after the enactment of restrictions aimed at limiting the spread of COVID-19, various local government and public health authorities around the world reported an increased sighting of rats. Such reports have yet to be empirically validated. Here we combined data from multi-catch rodent stations (providing data on rodent captures), rodent bait stations (providing data on rodent activity) and residents’ complaints to explore the effects of a six week lockdown period on rodent populations within the City of Sydney, Australia. The sampling interval encompassed October 2019 to July 2020 with lockdown defined as the interval from April 1st to May 15th, 2020. Rodent captures and activity (visits to bait stations) were stable prior to lockdown. Captures showed a rapid increase and then decline during the lockdown, while rodent visits to bait stations declined throughout this period. There were no changes in the frequency of complaints during lockdown relative to before and after lockdown. There was a non-directional change in the geographical distribution of indices of rodent abundance suggesting that rodents redistributed in response to resource scarcity. We hypothesize that lockdown measures initially resulted in increased rodent captures due to sudden shortage of human-derived food resources. Rodent visits to bait stations might not show this pattern due to the nature of the binary data collected, namely the presence or absence of a visit. Relocation of bait stations driven by pest management goals may also have affected the detection of any directional spatial effect. We conclude that the onset of COVID-19 may have disrupted commensal rodent populations, with possible implications for the future management of these ubiquitous urban indicator species.

## Introduction

Commensal rodents are abundant and pervasive pest species that cause vast damage to infrastructure, destroy crops, and spread diseases^1–5^. In urban areas, when a rodent pest becomes overabundant they contaminate food, damage infrastructure, increase risk of fire by gnawing on electrical wiring and pose a risk to public health as diseases carriers^2,5–14^. Annually, pest rodents cost billions of dollars in losses of food^15,16^. In many cases, pest rodents have become dependent on humans for food and harborage. Changes in human behaviors are known to affect commensal rodent populations^17^. Rodent abundance in cities is closely linked to socioeconomic conditions, accessibility to structures that offer nesting places, and human-derived food resources^17,18^. For these reasons, rodent control methods commonly include strategies to limit access to public garbage containers and potential nesting places^19^.

Previous studies indicate that changes in human behavior following natural catastrophes can result in changes in rat populations^20–22^. For example, large storms such as hurricanes, often cause large spikes in rodent abundance in urban environments^21,22^, perhaps due to a process known as counter-urbanization^23–25^. Natural disasters typically cause emigration of the urban human population which increases the abandonment of idle or degraded infrastructure and thus increases the availability of habitat for pest species^26^. For example, abandonment of urban environments in New Orleans following Hurricane Katrina appeared to drive an increase in the commensal rodent populations^25^. This increase in commensal rodent abundance can potentially increase the risk of zoonotic diseases transmission in the area (e.g. hantavirus disease and bartonellosis)^10–12^.

The appearance and rapid spread of SARS-CoV-2 in the human population during 2020 and 2021 is another form of natural disaster with profound effects on human behavior, particularly in urban environments. Some animal species may benefit from such a reduction in human activity, while others experience negative impacts from what has been called the “anthropause”^27^. The abrupt manifestation of COVID-19 in early 2020, forced governments around the world to enact preventative measures aimed at limiting the spread of SARS-CoV-2. These included mandatory social distancing, mandatory confinement to home (“lockdown”), and the closure or restricted operation of cafés, pubs, clubs, restaurants and supermarkets. Urban-dwelling species that are highly dependent on discarded or provided human food stuffs are expected to struggle under such conditions^27^. Thus, unsurprisingly, shortly after restrictions were enacted, reports from various local governments and public health authorities linked the closures of restaurants and food-related venues to increased sightings of rats^28–34^. Some of these reports described rodents engaging in atypical behaviors, such as rats being active during the day and in close proximity to humans^35–37^, as well as rats consuming conspecifics (e.g. muricide or cannibalism)^31,38^. A perceived increase in the abundance of rats is an unwelcome phenomenon for most city dwellers with adverse effects on the mental health of vulnerable individuals^39^. Given the multiplicity of significant implications for public health^7–9,35–37,40^, it is important to better understand how urban rodent populations have been impacted by COVID-19.

Parsons et al. released a preprint in which they investigated how stay-at-home measures affected rat sightings in various urbanized environments. They analyzed rat-related public complaints in New York City and Tokyo and surveyed pest control companies in the United States, Canada, Japan and Poland^41^. They reported that the two cities displayed a different patterns of rat-related public requests, with increases in Tokyo and decreases in New York City^41^. Importantly, they reported a positive association between rat sightings and food service establishments in both cities, with the formation of new rat sighting hotspots during the lockdown period^41^. Parsons et al. argued that the strong association between rat sightings and cafes or restaurants, as well as the development of new rat sightings hotspots, suggested mass movements of rats had been triggered by lockdown. Moreover, they suggest that this pattern was not observed in Warsaw, Poland due to the lack of clustering of restaurants^41^. An important limitation of Parsons et al.^41^ was that it used public perception as a proxy for rodent abundance and movements. Although there can be a reliable relationship between public complaints and rodent abundance^42^, this has not been validated during abnormal times such as COVID-19. It could be, for example, that public perceptions of rodent abundance are affected by cognitive and observational biases potentiated by COVID-19 restrictions and the increased use of social media^39,43,44^.

Here we aimed to more objectively determine how the COVID-19 pandemic restrictions affected pest rodent abundance over time within the City of Sydney Local Government Area, New South Wales, Australia. From January 2020, the New South Wales State Government enacted a series of preventative measures to limit the spread of COVID-19. These preventative measures included limits on the number of attendees at social gatherings, mandatory 14 days self-quarantine of all travelers entering Australia, mandatory closure of all non-essential businesses, restrictions for restaurant and cafes to only operate as “take-away”, as well as border closures with other Australian States and Territories. Similar to other parts of the world, shortly after these measurements were put in place, different media outlets started to anecdotally report an increase in rodent sightings^31–33^. The City of Sydney Council routinely traps and poisons rodents as part of their pest monitoring and control program and also compiles resident complaints. Here we used data from three sources to determine whether the enactment of COVID-19 preventative measures affected objective measures of the rodent pest population. These measures were (1) number of captures of rodents across up to 60 Flick SMART Multi-Catch rodent stations deployed by Council, (2) visitation by rodents at up to 942 bait stations deployed by Council, and (3) the number of rodent related residents’ complaints made to Council.

Given the unprecedented circumstances around COVID-19 restrictions, it is difficult to predict the exact effects they would have on urban pest rodent populations. Based on counter-urbanization studies^20–22, 24^, and recent hypotheses advanced by the scientific community^27^, we constructed the following predictions. We predicted that the pest rodent population inhabiting the City of Sydney Council would have suffered a significant reduction in numbers and activity during the COVID-19 restrictions, and that these effects might be reflected in the number of resident’s rodent related complaints received by the council during this period. We hypothesized that the anecdotal reports of increases in rodent sightings by media outlets^31,45,46^ might be a consequence of a reduction in food resources, in turn driving animals to engage in “bold” behaviors during foraging leading to increased captures and visits to bait stations^47–50^. Finally, based on Parsons et al.^41^, we predicted spatial shifts in rodent captures, activity, and rodent related complaints during lockdown.

## Results

### Trapping success

Results are reported across the three periods under consideration, namely Pre-lockdown (October 1st, 2019 to March 31st, 2020), Lockdown (April 1st to May 15th, 2020) and Post-Lockdown (May 16th to July 31st, 2020). The mean number of active Multi-Catch rodent stations per day during the entire sampling period (1st October 2019 to 31st July 2020) was 36.96 ± SE: 0.91 (Pre-lockdown 25.90 ± SE: 0.63; Lockdown 59.00 ± SE: 0.21; Post-lockdown 50.38 ± SE: 0.93). A total of 38 locations were sampled across all three periods; 22 locations were sampled during two of the three periods and only four were sampled during a single period.

**Figure 1:**
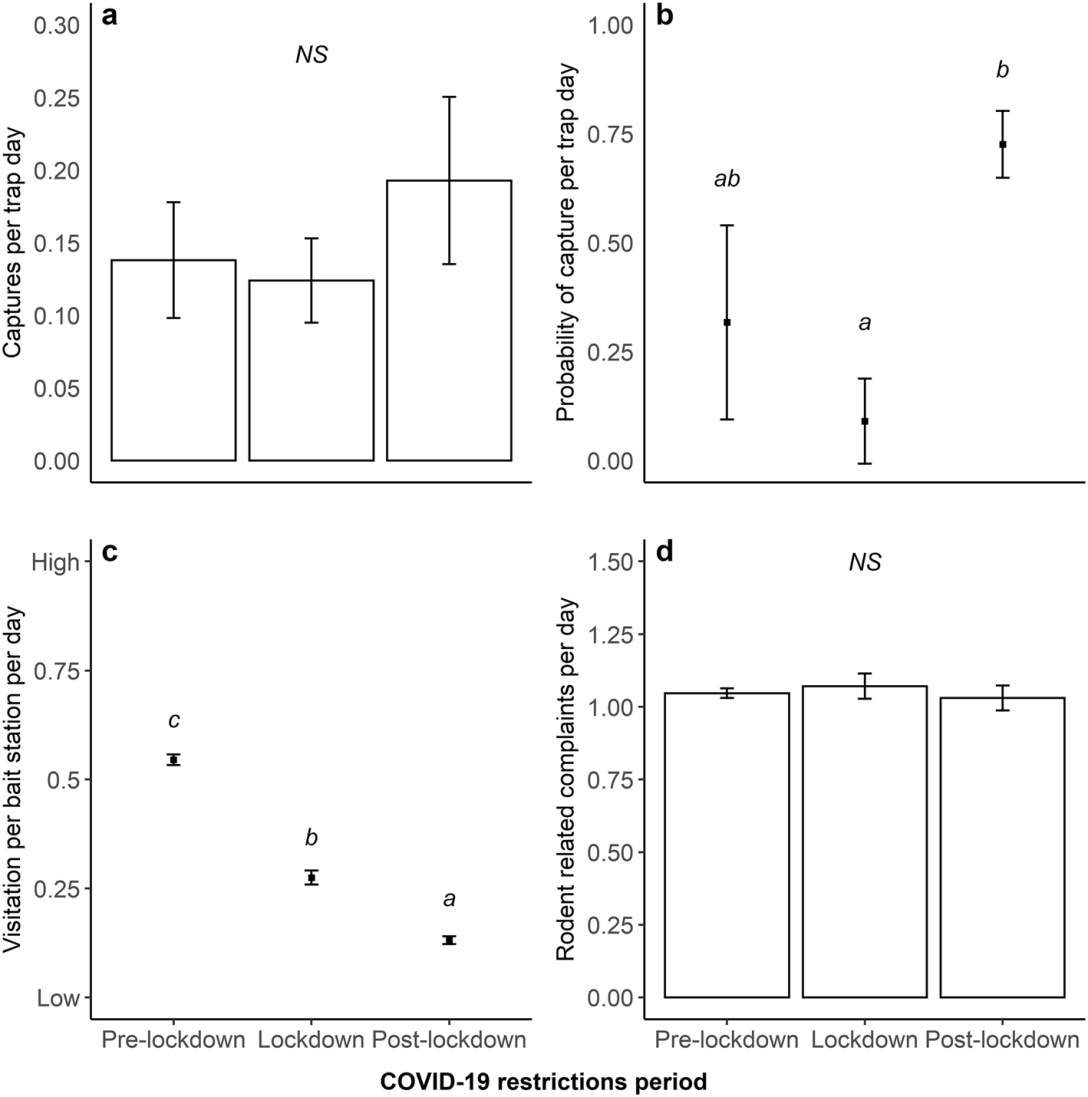
Rodent captures per trap day (**a**), probability of rodent captures per trap day (**b**), rodent visitation at bait stations (**c**) and rodent related residents’ complaints (**d**) recorded by the Council of the City of Sydney, prior during and post COVID-19 restrictions and social distancing measures imposed by the New South Wales State government (Mean ± SE). Superscripts represent Tukey-adjusted pairwise comparisons (α = 0.05).

A total of 851 rodents were captured during 305 days of deployment (mean: 2.79 ± SE: 0.469 per trap day). Overall, trapping success (i.e. number of captures and probability of capture) was affected by Period (i.e. Pre-lockdown, Lockdown and Post-lockdown) (*χ*^2^_2_ = 8.580; *P* = 0.014; Table 1). However, the overall number of rats caught per trap day did not differ between periods (Tukey’s HSD: Pre-lockdown vs Lockdown, *P* = 0.881; Pre-lockdown vs Post-lockdown, *P* = 0.288; and Lockdown vs Post-lockdown, *P* = 0.165; Table 1, Fig. 1a).

**Table 1:** Model summary, analysis of deviance (Wald Chi-squared tests) and Post-hoc Tukey adjusted pairwise comparisons for the model constructed to test trapping success per Multi-Catch Rodent station and day according to date and COVID-19 restriction period.

Conditional model fixed effects		Estimate	SE	z	*P*
(Intercept)		− 2.639	0.325	− 8.110	**< 0.0001**
Lockdown		4.497	2.286	1.967	**0.049**
Post-lockdown		− 5.133	2.372	− 2.164	**0.031**
Pre-lockdown (date)		− 0.001	0.002	− 0.523	0.601
Lockdown (date)		− 0.023	0.011	− 2.089	**0.037**
Post-lockdown (date)		0.020	0.009	2.274	**0.023**
**Zero-inflation model fixed effects**					
(Intercept)		− 2.013	1.326	− 1.518	0.129
Lockdown		35.800	11.520	3.108	**0.002**
Post-lockdown		− 2.771	3.715	− 0.746	0.456
Pre-lockdown (date)		0.004	0.006	0.700	0.484
Lockdown (date)		− 0.179	0.060	− 2.990	**0.003**
Post-lockdown (date)		0.019	0.013	1.410	0.158

Analysis of deviance table (Type III Wald chi-square tests)					
Fixed factors			d.f.	Wald-χ^2^	*P*
(Intercept)			1	65.778	**< 0.0001**
Period			2	8.580	**0.014**
Period (date)			3	9.810	**0.020**

Post-hoc Tukey adjusted pairwise comparison on the conditional model					
Period contrast	Ratio	SE	d.f.	t ratio	*P*
Pre-lockdown/lockdown	1.113	0.249	5251	0.480	0.881
Pre-lockdown/post-lockdown	0.716	0.159	5251	− 1.507	0.288
Lockdown/post-lockdown	0.643	0.156	5251	− 1.815	0.165

Post-hoc Tukey adjusted pairwise comparison on the zero-inflated model					
Period contrast	Odds ratio	SE	d.f.	t ratio	*P*
Pre-lockdown/lockdown	4.671	6.051	5251	1.190	0.459
Pre-lockdown/post-lockdown	0.176	0.149	5251	− 2.055	0.100
Lockdown/post-lockdown	0.038	0.043	5251	− 2.881	**0.011**

Model structure: Captures ~ Period + Period(Date) + offset (Log (Active Traps)) + Random (Location); Zero-inflation: ~ Period + Period(Date); Family: Negative Binomial. Data comprised daily captures from 20 to 60 multi-catch rodent stations deployed across the Council of the City of Sydney from October 2019 to July 2020. Bold values represent statistical significance (*P* < 0.05).

**Figure 2:**
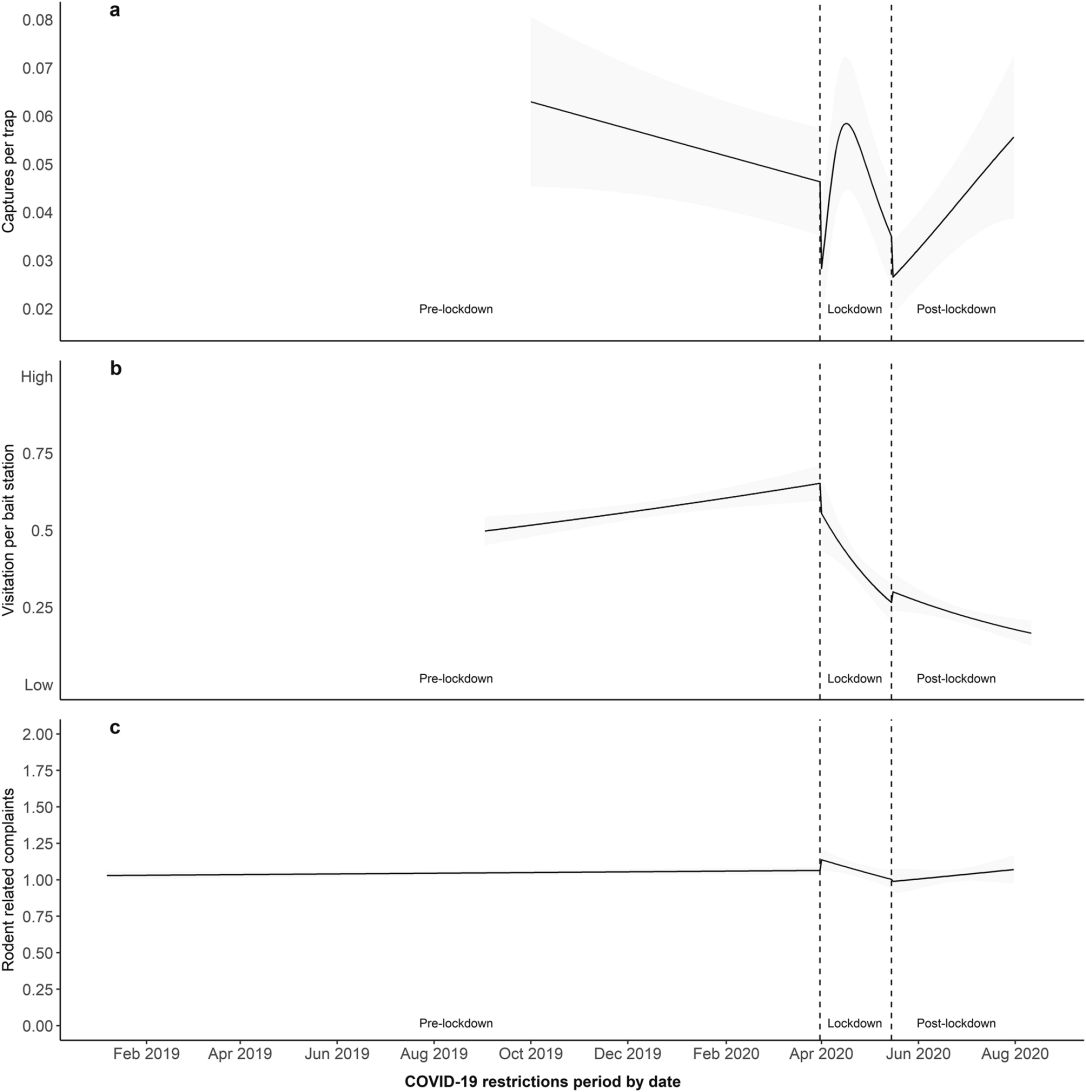
Estimated rodent captures per trap (**a**), rodent visitation per bait station (**b**), and rodent related complaints (**c**), received by the Council of the City of Sydney over time. The shaded grey area represents the standard error of the mean (SE). Dashed vertical lines represent the start and end points of COVID-19 restrictions and social distancing measures imposed by the New South Wales State Government.

The probability of capture per trap day was highest Post-lockdown and lowest during Lockdown (Tukey’s HSD: Pre-lockdown vs Lockdown, *P* = 0.459; Pre-lockdown vs Post-lockdown, *P* = 0.100; and Lockdown vs Post-lockdown, *P* = 0.011; Table 1, Fig. 1b). Trapping success was also affected by date within each period (*χ*^2^_3_ = 9.810; *P* = 0.020; Table 1). Captures per trap and probability of capture per trap remained stable over Pre-lockdown (Conditional model: *z* = − 0.523; *P* = 0.601; Zero-inflated model: *z* = 0.700; *P* = 0.484; Table 1, Fig. 2a); and decreased over time during Lockdown (Conditional model: *z* = 7.000; *P* = 0.037; Zero-inflated model: *z* = − 2.990; *P* = 0.003; Table 1, Fig. 2a). During Post-lockdown, only captures per trap, but not the probability of capture per trap, increased over time (Conditional model: *z* = 2.274; *P* = 0.023; Zero-inflated model: z = 1.410; *P* = 0.158; Table 1, Fig. 2a).

### Rodent visitation to bait stations

Results are reported across the three periods under consideration, namely Pre-lockdown (October 2nd, 2019 to March 31st, 2020), Lockdown (April 1st to May 15th, 2020) and Post-Lockdown (May 16th to August 11th, 2020). Note the sampling period is slightly longer for bait station data than for trapping data. The bait stations employed by council provided only binary data on whether there had been a visit during the intervening period since the station was last checked. With routine bait station relocations by the Council’s pest management contractors, a total of 661 unique locations were recorded during the entire sampling period. The mean number of rodent bait stations checked per day during the entire sampling period was 51.45 ± SE: 1.68 (54.66 ± SE: 2.05 Pre-lockdown; 41.30 ± SE: 3.93 during Lockdown; and 45.18 ± SE: 4.01 Post-lockdown). There were 376 locations sampled across all three periods; 50 locations were sampled only during two of the three periods and 235 were sampled during only a single period. Average rodent visitation per bait station per day, over 345 days of deployment was recorded as 0.516 ± SE: 0.052 (0 = Not visited and 1 = Visited).

Rodent visitation per bait station per day was affected by period (*χ*^2^_2_ = 46.502; *P* < 0.0001; Table 2). The highest visitation per bait station per day was recorded Pre-lockdown (Tukey’s HSD, *P* < 0.0001, Table 2, Fig. 1c), the lowest Post-lockdown (Tukey’s HSD, *P* < 0.0001, Table 2, Fig. 1c), with visitation per bait station per day during Lockdown at an intermediate level (Tukey’s HSD, *P* < 0.0001, Table 2, Fig. 1c). Rodent visitation was also affected by date within each period (*χ*^2^_3_ = 68.014; *P* < 0.0001; Table 2). Pre-lockdown, rodent visitation per bait station remained stable over time (*z* = 1.724; *P* = 0.085; Table 2, Fig. 2b); and decreased over time during Lockdown and Post-lockdown (*z* = − 7.639; *P* < 0.0001; and *z* = − 2.624; *P* = 0.009, respectively; Table 2, Fig. 2b).

**Table 2:** Model summary, Analysis of Deviance (Wald Chi-squared tests) and Post-hoc Tukey adjusted pairwise comparisons for the model constructed to test rodent activity scores from bait stations according to date and COVID-19 restriction period.

Fixed effects	Estimate	SE		z	*P*
(Intercept)	0.105	0.067		1.571	0.116
Lockdown	8.539	1.252		6.819	**< 0.0001**
Post-lockdown	− 0.003	0.760		− 0.004	0.997
Pre-lockdown (date)	0.001	0.000		1.724	0.085
Lockdown (date)	− 0.041	0.005		− 7.639	**< 0.0001**
Post-lockdown (date)	− 0.007	0.003		− 2.624	**0.009**

Analysis of deviance table (Type III Wald Chi-square tests)					
Fixed factors			d.f	Wald-χ^2^	*P*
(Intercept)			1	2.469	0.116
Period			2	46.502	**< 0.0001**
Period (date)			3	68.014	**< 0.0001**

Post-hoc Tukey adjusted pairwise comparison					
Period contrast	Odds ratio	SE	d.f.	z ratio	*P*
Pre-lockdown/lockdown	3.160	0.234	Inf	15.571	**< 0.0001**
Pre-lockdown/post-lockdown	7.950	0.580	Inf	28.447	**< 0.0001**
Lockdown/post lockdown	2.510	0.234	Inf	9.925	**< 0.0001**

Model structure: Activity Score ~ Period + Period (Date) + Random (Location); Family: Binomial. Data comprised rodent activity score (i.e. low = 0 or high = 1) from 942 bait stations deployed across the Council of the City of Sydney from September 2019 to August 2020. Bold values represent statistical significance (*P* < 0.05).

### Rodent related residents’ complaints

Residents’ complaints are reported across the three periods under consideration, namely Pre-lockdown (January 7th, 2019 to March 31st, 2020), Lockdown (April 1st to May 15th, 2020) and Post-Lockdown (May 16th to July 31st, 2020). Note the sampling period is slight longer than for both bait station and trapping data. The City of Sydney Council received 242 rodent related complaints during the entire interval under consideration; mean: 0.423 ± SE: 0.031 complaints per day. There were 161 (66.5%) complaints received through the Councils website, 72 (29.8%) through emails, and 9 (3.7%) by phone. Across the 33 suburbs and 23 localities within the Council, complaints were received from only 22 suburbs (Pre-lockdown 18 suburbs; Lockdown 10 suburbs; Post-lockdown 12 suburbs). Complaints were received from five suburbs during all three periods, eight suburbs during two periods and nine suburbs during a single period. The number of complaints per day received by the Council was not affected by period (*χ*^2^_2_ = 1.207; *P* = 0.547; Table 3, Fig. 1d) or date within period (*χ*^2^_3_ = 1.612; *P* = 0.657; Table 3, Fig. 2c).

**Table 3:** Model summary, Analysis of Deviance (Wald Chi-squared tests) and Post-hoc Tukey adjusted pairwise comparisons for the model constructed to test number of rodent related residents’ complaints, received by the Council of the City of Sydney, according to date and COVID-19 restriction period.

Fixed effects	Estimate	SE	d.f	t value	*P*
(Intercept)	0.029	0.030	224	0.966	0.335
Lockdown	1.389	1.410	224	0.985	0.326
Post-lockdown	− 0.556	1.144	224	− 0.486	0.628
Pre-lockdown (date)	0.000	0.000	224	0.677	0.499
Lockdown (date)	− 0.003	0.003	224	− 0.957	0.340
Post-lockdown (date)	0.001	0.002	224	0.488	0.626

Analysis of deviance table (Type III Wald Chi-square tests)					
Fixed factors			d.f	Wald-χ^2^	*P*
(Intercept)			1	0.933	0.334
Period			2	1.207	0.547
Period (date)			3	1.612	0.657

Model structure: Log (Complaints) ~ Period + Period (Date) + Random (Suburb); Family: Gaussian. Data comprised the number of rodent related residents’ complaints received by the Council from January 2019 to August 2020.

### Spatial analysis of trapping success, rodent visitation and residents’ complaints

**Figure 3:**
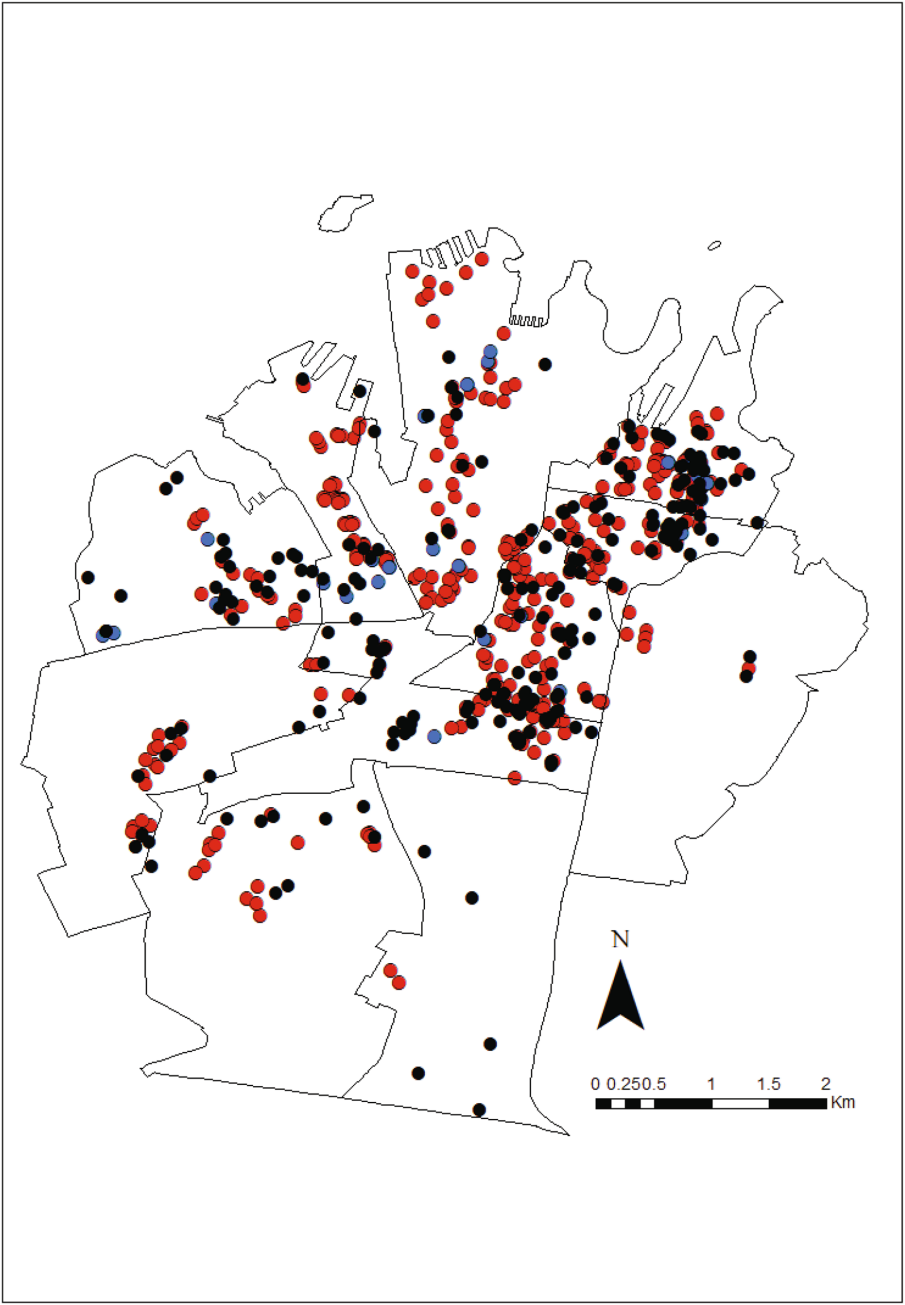
Locations of multi-catch rodent stations (), rodent bait stations () and residents’ rodent complaints () within the Council of the City of Sydney. The eleven Statistical Area 2 (SA2) which make up the City of Sydney are shown. Map generated using ArcGIS Desktop v10.5^88^.

**Figure 4:**
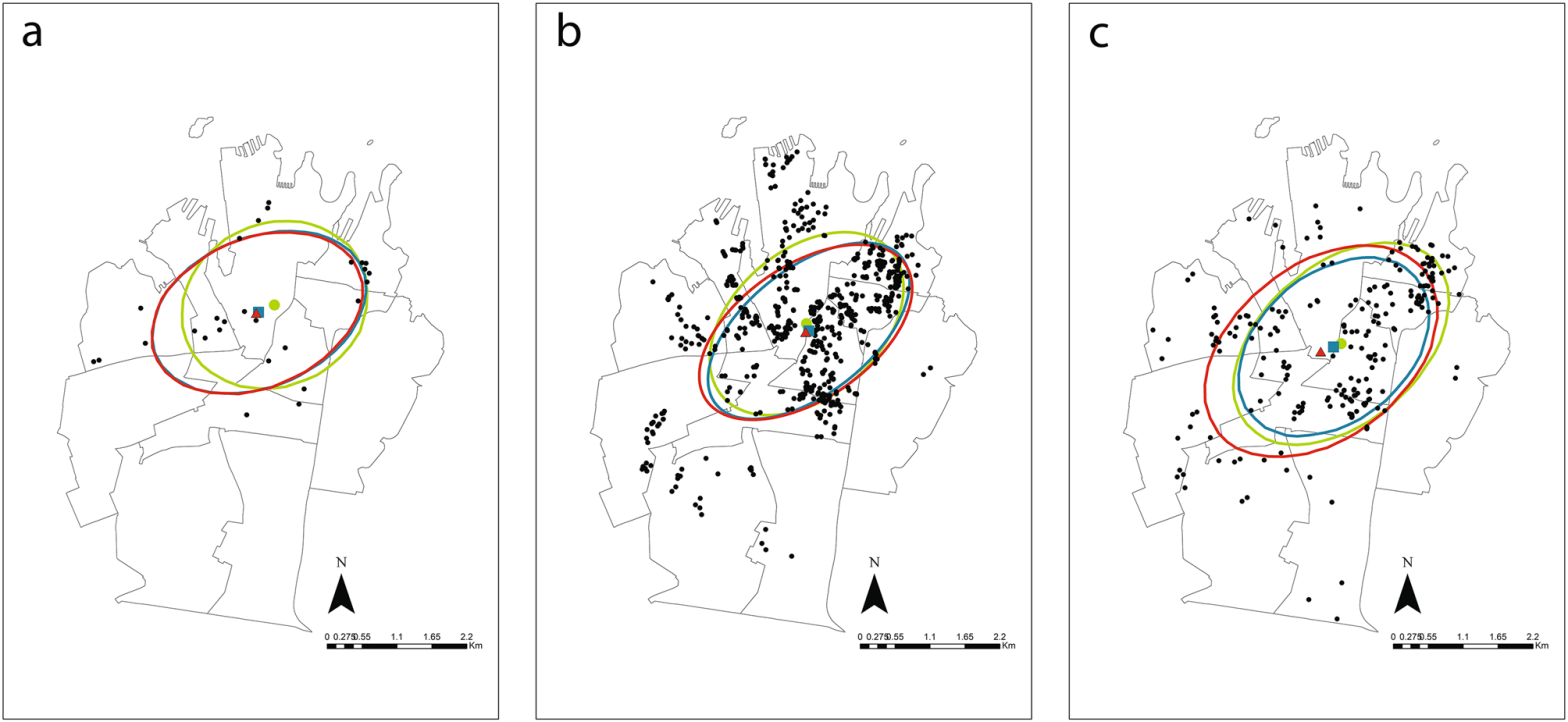
Distribution of trapping success (**a**), rodent bait stations visitation (**b**) and residents’ rodent complaints (**c**) within the Council of the City of Sydney and their mean centers and associated directional ellipses during pre-lockdown (), lockdown () and post-lockdown () periods. Maps generated using ArcGIS Desktop v10.5^88^.

Multi-Catch rodent stations were operated within seven of the eleven Statistical Areas Level 2 (SA2) that contain the Council, whereas rodent bait stations were operated in all eleven SA2. Rodent related residents’ complaints were made in all SA2 (Fig. 3). For all three measures of rodent activity (trapping, bait station visitation and complaints) their mean centers and directional ellipses were approximately equivalent during the Pre-lockdown, Lockdown and Post-lockdown periods (Fig. 4a–c). The spatial distribution of trapping success did not differ (i.e. significant correlation) between Pre- versus Post-lockdown periods (r_SP_ 0.829, *P* = 0.021), but was different (i.e. correlation not significant) during Lockdown relative to both Pre- (r_SP_ 0.270, *P* = 0.558) and Post-lockdown (r_SP_ 0.679, *P* = 0.094). The spatial distribution of rodent visitation differed (i.e. correlation not significant) across all periods: Pre-lockdown relative to Lockdown (r_SP_ 0.582, *P* = 0.060) and Post-lockdown (r_SP_ 0.591, *P* = 0.056); and Lockdown relative to Post-lockdown (r_SP_ 0.518, *P* = 0.102). The spatial distribution of rodent related residents’ complaints did not change (i.e. significant correlation) from Pre-lockdown relative to both Lockdown (r_SP_ 0.791, *P* = 0.004) and Post-lockdown (r_SP_ 0.709, *P* = 0.015) periods. However, the spatial distribution of rodent related residents’ complaints did change (i.e. correlation not significant) from Lockdown to Post-lockdown (r_SP_ 0.431, *P* = 0.186). Finally, the spatial distribution of the three measures (trapping, bait station visitation and complaints) were not correlated, except for trapping success and rodent visitation during Pre-lockdown (r_SP_ − 0.775, *P* = 0.041).

## Discussion

The current study used datasets supplied by the City of Sydney Council to examine the impact of a 6-week lockdown on three different measures of rodent activity, namely rodent captures, bait station visitation and resident complaints. The general trends for these three measures differed quite markedly (Tables 1, 2 and 3; Fig. 1). All three measurements were quite stable prior to COVID-19 restrictions (Fig. 2) but the introduction of Lockdown seemed to reduce captures and bait station visitation. These two measures then separated Post-lockdown with rodent captures increasing while bait station visitation continued to decrease (Tables 1 and 2; Fig. 2a,b). Interestingly, the dynamic relationship between rodent captures and bait station visitation was not due to actual number of rodents captured per trap day, which remained the same between periods (Table 1; Fig. 1a). Instead, this relationship was based on the probability of capture per trap day (Table 1; Fig. 1b,c). These might be due to the high variability in the measures across the duration of each period, shown by the over time effects detected within each period (Tables 1, 2; Fig. 2), as well as the nature of the measurement and our analyses.

Due to the over representation of zeros and overdispersion of the Multi-Catch rodent station data, the use of a Zero-inflated model was warranted^51,52^. This type of model allowed us to detect any effects of COVID-19 restrictions on the number of rats caught in a particular trap per day, as well as the probability of capture of at least one rat in a given day. These two measurements might differ under certain circumstances. For example, if a small proportion of traps record high capture rates, while most record no captures, the mean number of captures per trap night might be the same as if most traps recorded a number of captures closer to the mean, and just a minority recorded no captures. Meanwhile, in this same example, the probability of capture would be low in the first scenario, since most traps did not record captures, and higher in the second scenario, since most traps recorded at least one capture. This might explain the patterns we described across all three periods (i.e. Pre-lockdown, Lockdown and Post-lockdown) where the number of rats captured per trap per day did not change, but the probability of capture did (Table 1; Fig. 1a). Further supporting this idea is our spatial distribution analyses, where we recorded a slight change in the spatial distribution of rodent captures during Lockdown in comparison to Pre-lockdown and Post-lockdown (Fig. 4a), which seems to suggest that some traps recorded higher trapping rates, while most recorded very low trapping rates.

With the exception of rodent captures, our findings seem to contradict observations of rodent population after hurricanes^21,22^. We hypothesize that this might be due to the undeniable temporal and physical differences between a climatic event and a pandemic. Climatic events are short-lived, with vast physical effects on the landscape, shifts in habitat characteristics, and increasing landscape heterogeneity^2,53^. In contrast, social restrictions were limited to closure of restaurants, cafes and other food venues^54^, causing a reduction in human-derived food resources where they have been plentiful before.

Rodent captures per trap showed an abrupt spike during Lockdown, followed by a steep and rapid decline and a slow recovery during Post-lockdown (Table 1, Fig. 2a). In contrast, Lockdown seemed to have triggered a steep decline in rodent activity that continued Post-lockdown (Table 2, Fig. 2b). We found no temporal changes in the number of rodent related complaints received by the council (Table 3, Fig. 4). We found no directional spatial changes for any of the measurements between Pre-lockdown, Lockdown and Post-lockdown periods (Fig. 4). However, the distribution of trapping success and rodent visitation at bait stations Pre-lockdown appeared spatially related, but this relationship was disrupted during Lockdown and continued to be disrupted Post-lockdown. Since the spatial distribution of trapping success only changed during Lockdown, relative to Pre- and Post-lockdown spatial distributions, the disruption in the relationship between trapping success and rodent visitation seems to be due to the change in spatial distribution in rodent visitation during Post-lockdown. Rodent visitation spatial distribution changing across all periods, seems to suggest that this measure of rodent abundance did not recover from the disruption suffered during Lockdown.

Commensal rodents show high levels of neophobia^55,56^ and taste aversion^2,53^ resulting in high levels of ‘trap-shyness’^57,58^ and low bait acceptance^59,60^. However, a reduction in food resources might have driven animals to engage in “bold” behaviors during foraging whilst in a lower physiological state^61,62^. These hunger-driven behaviors might explain reports of rats feeding in close proximity to humans^31,45,46^ as well cannibalism^31,38^. Ultimately, hunger might have caused these animals to overcome their neophobia and taste aversion, resulting in a decrease in trap shyness potentially driving an increase in mortality by electrocution. However, under this premise, we expected that rodent activity at bait station would reflect captures and spike, yet we did not find that. One possibility is that the reduction in food resources was not enough for animals to overcome their taste aversion towards the commonly used rodenticides at the bait station. In contrast, Multi-Catch rodent stations are baited with non-toxic food attractants, thus taste aversion might be unlikely. Another possibility is the difference in resolution of the data between Multi-Catch rodent stations and bait stations. Rodent visitation at bait station was recorded as “not visited” or “visited”, collapsing the potential variability present in rodent visitation in a binary measure. Thus, a record for a peak in visitation (e.g. increase signs of rodents and higher levels of bait taken from the bait stations) could have effectively being lost by grouping them at the same level as reduced, yet present, visitation (e.g. limited signs of rodents and bait taken). However, this does not explain why rodent related residents’ complaints did not change between periods or over time within each period.

Rodent related residents’ complaints seem to be completely disassociated from rodent captures and activity, remaining stable over time and between periods, except for a change in spatial distribution observed Post-lockdown. This is contrary to what has been reported during periods of no disturbance^42^; and during COVID-19^41^. One possible explanation is that, during the Lockdown period, most people remained home social distancing, with many leaving the Council to neighboring areas, similar to counter-urbanizations^20^. With less people within the Council, the chances of rats sightings would have been reduced, thus the lack of change in the number of rodent related complaints might not reflect the rodent population, but might be an artefact of less people commuting and occupying public spaces. Furthermore, a decrease in resources by the closure of restaurants and cafes, might have driven rodents to move from public spaces to private residences where food waste was still available. People that then experienced an increased in rodents’ sightings within their residence, might have contacted pest controllers directly and not the Council. Similarly, prior to COVID-19 restrictions, a single rat sighted around a restaurant café or public space, could have been reported by a high number of customers and residents. In contrast, during the Lockdown period, several rats sighted within a resident would incite a single complaint to the Council. Previous research on the relationship between the number of rodent related residents’ complaints and the actual rodent population, show a direct relationship between the two^42^, but do not account for disruptions in human behavior such as the ones brought up by COVID-19. More complex responses in rodent related residents’ complaints, in relationship with COVID-19, have been reported recently^41^ and are in concordance with our findings.

A higher mortality due to a decrease in carrying capacity might explain the activity decline that we found during the lockdown period and in captures later on during the same period^63,64^. It is highly unlikely that the peaks in rodent captures would be due to an increase in the rodent population, given that the lockdown period lasted only 45 days. It is also important to note that we classified lockdown as the short period during which COVID-19 restrictions were at their peak, with cancellations of all social gatherings, mandatory closure of all non-essential businesses including restaurant and cafes^54^. Both the pre-lockdown and the post-lockdown periods encompassed the gradual increase and decrease of these restrictions over several weeks^54^. Therefore, even rats, which are known for their prolific reproduction^65,66^, would not be able to reproduce and mature in such a limited time frame and even less with disruptions on human behavior still present. The recovery in the population is therefore expected to be more gradual, like the steady but slow increase in rodent captures we found in the post-lockdown period. Interestingly, rodent activity again did not mirror the rodent captures data post-lockdown. As argued above, this difference might be a consequence of the nature of the data collected. If the peak in rodent activity was indeed due to abnormal foraging behavior caused by starvation followed by a population reduction, it is highly possible that the lockdown acted as a genetic bottleneck. Previous research has shown that, after such a mortality event, the genetic variation within the remaining population could be up to 90% lower than the original population^67^.

Given that our data did not cover several years, we were unable to account for natural seasonal cycles in the rodent population. Several studies have shown that urban rodent populations follow a seasonal gradient that reflect both human changes in behaviors and temperature^17,42,68,69^. Colder months seem to trigger lower rodent activity, that then increase towards spring and peaks in summer^42,68,69^. During harsh winters, rats have been reported to move from outdoors to indoors in search of food and warmth^70^, and this behavior has been linked to an increase in rodent related resident’s complaints^17,71^. Our Pre and Post-lockdown multi-catch station data seems to support this, but not so our bait station data. However, the lowest temperatures recorded during this study, were recorded in July during Post-lockdown (i.e. 7 °C^72^), when trapping success was not the lowest and was instead steadily increasing. It is possible that in a subtropical City such as Sydney (average minimum temperature 15.7 °C^72^) the effect of seasonal changes in temperature might not be as strong as that detected in laboratory studies^68,69^ and more temperate cities^42^. Additionally, it has been well documented that cities are “heat islands” that experience significantly milder winters than surrounding areas^73^. This might be more pronounced in coastal cities like Sydney. Seasonal changes in rat activity have also been linked to changes in human behavior, and not solely a respond to the change in temperature, with people spending more time outdoors during the warmer weather, thus increasing sightings and resource availability^17,42^. Thus, the abrupt reduction in human activity during the lockdown, might have acted as an early onset of winter. Moreover, the expected seasonal changes in rodent activity cannot explain the abrupt increase and decline in captures, nor the accelerated decline in rodent visitation during Lockdown. Therefore, we argue that the effects we report are solely due to the changes in human behaviors, and unintended effects on the rodent population, elicited by the COVID-19 restrictions.

We found no evidence of directional spatial changes driven by the Lockdown. This supports the findings Parsons et al. reported from Warsaw, Poland but contrast with their results from New York City and Tokyo^41^. They suggested that COVID-19 Lockdown measures trigger an increase in rodent movement and potential massive migrations, based on the increased association of rats and food service establishments and the formation of new hotspots of rat sightings in New York City^41^. However, the level of rodent migration Parsons et al.^41^ suggests—i.e. tenths of kilometers—is difficult to reconcile base on the well-known site fidelity pest rodents species show^74^. However, this does not negate the possibility that rodents might have move from areas of normally abundant resources that were suddenly reduced—i.e. restaurants and cafes—to areas where some limited level of resources remained—e.g. private residences. In fact, although we did not detect any directional spatial changes, we did detect changes in the spatial distributions of our rodent abundance measures. Parsons et al.^41^, suggest that the pattern of movement reported, was not recorded in Warsaw, potentially because of the lack of restaurant clusters in that city, a situation that may be similar to the one in Sydney. The City of Sydney Council is not comprised by distinct clusters of offices and residents, with most buildings being of “dual use”, with business at ground floor and residential apartments above. Without distinct residential and business suburbs within the City of Sydney Council, rats might move very short distances to small pockets within the surrounding area in search of resources, thus long-distance directional migration would not be detected. Moreover, is crucial to recognize the limitations of the data we used. Both Multi-Catch and bait rodent stations were sometimes removed or relocated to target “problem” areas within the Council based of the Council’s pest management objectives. This process might have removed any spatial effect. Therefore, although we did not detect any obvious directional shifts in spatial distribution, if such shifts did occur, they were most likely subtle, and we were unable to detect them.

Although the risks of commensal rodents to be infected or transmit SARS-CoV-2 are low^75^, we know that these animals pose other health risks^2,7–14^. Thus, an increase in rodent-human interactions has the potential to place further strains on health systems around the world. Fortunately, our data seems to suggest the increase in rodent–human interactions, as reported by the media, might be an overstatement. Undoubtedly, the onset of COVID-19 might have disrupted not only human behavior, but also commensal rodent populations, yet the implications for the future management of these species is still uncertain. The disruptions caused by COVID-19 restrictions to the rodent population seem to differ depending on the measured used. Our data illustrates how two different control methods (i.e. lethal traps and bait stations) can show similar patterns, and potentially have similar efficacy, during normal conditions. However, an abrupt change in human behavior can disrupt this efficacy and possibly change the characteristics of the individuals targeted by each method. Although it is too early to determine if these disruptions would have long term effects on management outcomes, the different patterns reported for lethal traps and bait stations highlights the need to follow integrated pest management (IPM) frameworks that do not rely on a single control methods^76^.

## Materials and methods

### Study area

The City of Sydney Council (hereafter Council) is the largest city, by population, in Australia, with 240,229 residents^77^. It comprises a highly urbanized part of the Greater Sydney region and includes the Central Business District of Sydney and many of Sydney’s major tourist attractions (e.g. the Opera House). It borders Port Jackson in the north, the Woollahra Municipal Council area and Randwick City in the east, the Bayside Council area in the south, and the Inner West Council area in the west^77^. Sydney has a humid subtropical climate (average maximum = 21.3 °C; average minimum = 15.7 °C;^72^. The Council is composed of 33 suburbs and 23 localities. In Australia, suburbs are defined as geographical subdivisions used for address purposes and refer to portions of a city that can include inner-city, outer-metropolitan, and industrial areas. The Australian definition of suburb is more alike the American and British definitions of “district” or “neighborhood”. Localities are defined as historically unrecognized geographical units that due to common informal used, and later government decree; official boundaries were established and are currently recognized geographical features.

### Data sourcing

All data used in this research were obtained from the Council. As part of Council’s ongoing rodent control operations, pest management contractors have deployed multi-catch rodent stations as well as rodent bait stations across the Council. The Council conducts pest management operations only on Council property and outdoors. Rodent captures and activity are recorded regularly, and the data are stored by the Council. The Council also receives residents’ complaints through phone calls, emails or through electronic complaint forms found on the Council’s website. These complaints are compiled and stored by the Council.

Rodent pest management by the Council targets brown rats (*Rattus norvegicus*)*,* and black rats (*R. rattus)*; the former being the most common species found in the urban environment. Common house mice (*Mus musculus*) are also found within the council, but they seldom occur outside of buildings, and their control is considered the responsibility of property owners and managers. Due to unreliable identification by pest management contractors and general public, the records used for this study do not include species specific data (i.e. *R. rattus* vs *R. norvegicus*). A subsample of 95 rat carcasses obtained from live trapping and multi-catch rodent stations across the Council suggests *R. norvegicus* to be the dominant species within the council (i.e. 88 *R. norvegicus* and 7 *R. rattus).*

### Multi-catch rodent stations dataset

Flick SMART Multi-Catch rodent station is an internationally patented rodent trap design^78^. This trap consists of a trigger mechanism that kills the animal by an electric current. The trap has a built-in programmable computer with a SIM card, enabling it to communicate via the mobile network when it has been activated. This trap can catch multiple animals (eight maximum), before it needs to be serviced.

Under a pest management contract between the Council and Flick Anticimex Pty Ltd, 20–60 multi-catch rodent stations were deployed outdoors on public places and Council own land from October 2019 to July 2020. Deployment was non-random and guided by strategic pest management priority zoning. Multi-catch rodent stations were sometimes removed or relocated, whenever they were damaged or if trapping success did not reflect the Council’s pest management goals (i.e. zero captures for 4 weeks). These stations were baited with barbeque grease and commercially available rodent attractants The Council received monthly reports from Flick Anticimex Pty Ltd over the deployment interval that contained the number of traps active, the location in latitude and longitude coordinates of each station and the number of captures per day per each trap.

### Rodent bait stations dataset

Additionally, 942 bait stations (PROTECTA EVO Ambush and PROTECTA LP, Bell Laboratories, Inc.) were deployed outdoors on public places and Council own land from September 2019 to August 2020. Deployment was non-random and guided by strategic pest management priority zoning. Bait stations were sometimes removed or relocated, whenever they were damaged or if rodent activity did not reflect the Council’s pest management goals (i.e. low to no activity for 4 weeks). These stations were baited with commercial poisoned baits (Bromadiolone: Contrac Blox and Contrac Soft Bait; Brodifacoum: Ditrac Blox; Difethialone: Generation Block and Rodilon Soft Block; Flocoumafen: Storm Secure Block and Storm Soft Bait). Baits were randomly rotated at each station, to prevent rodents developing aversion to any bait. Each station was checked regularly (mean: 10.56 Days ± SE: 0.06) and scored according to the rodent visitation i.e. low: no bait consumed and no visual signs; or high: bait consumed and visual signs present. Following strategic pest management principles, bait station with consistent low activity score were relocated. The location for each bait station was recorded in UTM coordinates to facilitate spatial analyses.

### Rodent related residents’ complaints dataset

Murray, et al.^42^ previously showed that the number of rodent related residents’ complaints can be used as an estimate of rat relative abundance across diverse urban landscapes. Later Parsons et al.^41^ used rat-related public complaints and surveyed pest control companies to estimate changes in rodent pest populations during COVID-19 restrictions. However, the relationship between rodent related residents’ complaints has not been validated during disruptions in human behavior such as the ones created by the COVID-19 pandemic. Therefore, we accessed all rodent related complaints made to the Council from January 2019 to August 2020 and included these data in our analyses.

Complaints were received through phone calls, emails or through electronic complaint forms found on the Council’s website. All complaints contained the date and street address. Identifying information was removed from the complaint dataset, with street address transformed to UTM coordinates to facilitate spatial analyses, and suburb used for statistical analyses.

### COVID-19 pandemic restrictions

Following the rapid increase in COVID-19 cases at the beginning of 2020, the New South Wales State Government enacted a series of preventative measures to limit the spread of the disease. These preventative measures included limits in the number of attendees at social gatherings, mandatory closure of all non-essential businesses, restrictions to only operate as “take-away” for restaurant and cafes, and border closures with other Australian States and Territories. We used the publicly available timeline of these measures^54^, to classify the datasets into three “Periods”. *Pre-lockdown* was defined as the period prior to March 31st, 2020; the *Lockdown* was defined as the period from the April 1st to May 15th, 2020; and *Post-lockdown* was defined as the period from May 16th onwards.

### Statistical analyses

To explore whether there was any effect of COVID-19 restrictions overall on pest rodent population and residents’ perception within the Council, we performed statistical analyses in R 4.0.2^79^. Each dataset had a different distribution and were at slightly different spatial scales, hence the need for different models.

Due to the over representation of zeros and overdispersion, the Multi-Catch rodent station dataset was analyzed by a Zero-inflated Generalized Linear Mixed Model (GLMM) using the function *glmmTMB* from the package “glmmTMB” version 1.0.2.1^80^ with a negative binomial error distribution. Zero-inflated models are used for count data that are over dispersed and are characterized by an excess of zeros^51,52^. The data distribution used for zero-inflated models combines the negative binomial or Poisson error distributions and the logit error distribution functions, effectively modelling count values separately from the excess zeros which are treated as binomial data (i.e. 0 or 1)^51,52^. Therefore, model outputs for zero-inflated models include analyses of count data, in our case number of animals captured, and binomial data, in our case probability of capture. The rodent bait station dataset was analyzed by a GLMM with a binomial error distribution, using the function *glmer* from the package “lme4” version 1.1.23^81^. The rodent related residents’ complaints dataset was analyzed by another GLMM, using the functions *lmer,* from the package “lme4” version 1.1.23^81^. With the exception of the models constructed to test the rodent bait station dataset, residual plots and the Pearson’s dispersion test were used to identify the best distribution and link for each model^82^.

Each model aimed to test the effects of COVID-19 restrictions on the number of rodent captures per day (Multi-Catch rodent stations), the level of rodent visitation (Rodent bait stations) and residents’ reporting of rodent activity (number of complaints). Period (i.e. Pre-lockdown, Lockdown and Post-lockdown), numerical date (day as integer starting at 1 to last sampling day per data set, when each data point was collected), and numerical date nested within period (as each date value only occurred within a particular period) were included as fixed factors. The model testing the number of rodent related residents’ complaints initially included the mean of communication (i.e. email, council website or phone call) as a covariate, due to lack of significance (*P* > 0.50) this covariate was later removed in order to simplify the model^83^.

To account for intrinsic differences in rodent captures, visitation and number of complaints based on location, each model included location (i.e. trap or bait station coordinates, or the suburb where complaint was received from) as the only random factor. Additionally, in the case of the Multi-Catch rodent station and rodent bait stations, to account for differences in sampling effort due to variable number of stations deployed across the sampling period, the number of active Multi-Catch rodent station or bait stations checked per day were included in the models as offsets.

To generate *P*-values, Wald Chi-square tests were applied to all models using the function *Anova* from the package “car” version 3.0.9^84^. Post-hoc pairwise comparisons with Tukey adjustments were carried out by the functions *emmeans*, and *pairs* from the package “emmeans” version 1.5.0^85^, and the function *cld* from the package “multcomp” version 1.4.13^86^. Graphs were constructed using package “ggplot2” version 3.3.2^87^.

The spatial distribution of total rodent captures (Multi-Catch rodent stations), the level of rodent visitation (Rodent bait stations with high visitation) and residents’ perceptions of rodent activity (total number of complaints) were visualized by creating dot maps based on latitude and longitude or UTM and the Inner Sydney and Eastern Suburbs-North polygons of the Statistical Areas Level 3 (SA3) dataset set 2016 (Geographic Datum of Australia 1994). Data were projected in UTM 56S using ArcGIS 10.5^88^. For each dataset overall and for the three study periods, mean centers and directional ellipses were calculated (Spatial Statistics. ArcGIS 10.5. ESRI). Rodent captures, visitation and complaints data were summed to SA2 areas: the 10 areas which make up Inner Sydney SA3 and the adjacent Paddington-Moore Park SA2 which lies within SA3 Eastern Suburbs-North. The correlations (r_SP_) between these data by SA2 were calculated on SPSS v24^89^.

## Data Availability

Most of the data that supports the findings of this study are available from Dryad^90^ but restrictions apply to the availability of the rodent related residents’ complaints data, which were used under license for the current study, and so are not publicly available. Data are however available from the authors upon reasonable request and with permission of the Council of the City of Sydney.
